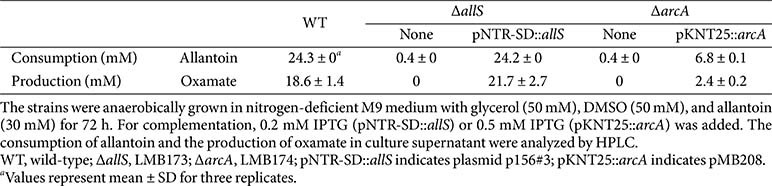# Dual Regulation of the *allF* Operon by ArcA and AllS Enables Anaerobic Allantoin Utilization in *Escherichia coli*

**DOI:** 10.4014/jmb.2507.07057

**Published:** 2025-09-24

**Authors:** Nam Yeun Kim, Ok Bin Kim

**Affiliations:** 1 Division of EcoScience, Ewha Womans University, Seoul 03760, Republic of Korea; 2 Department of Life Science, Ewha Womans University, Seoul 03760, Republic of Korea

**Keywords:** *allF* operon, ArcA, anaerobiosis, AllS, allantoin, *Escherichia coli*

## Abstract

During anaerobic growth, *Escherichia coli* is capable of utilizing allantoin as its sole nitrogen source. Allantoin, a purine derivative, is initially degraded into ureidoglycolate with the release of two NH_3_. Ureidoglycolate can then enter either the glycerate branch (to 2-phosphoglycerate) or the oxamate branch (releasing additional NH_3_). In the oxamate branch, ureidoglycolate first is oxidized to oxalurate, then converted to oxamate and carbamoyl phosphate by oxamic transcarbamylase (OXTCase); carbamoyl phosphate, in turn is used by carbamate kinase (CK) to generate ATP and NH_3_. This study focuses on the transcriptional regulation of OXTCase and CK, which catalyze the final two steps of the oxamate branch and are encoded by the *allFGHK* operon (*allF* operon), the most recently identified genes in the allantoin pathway. Transcription of the *allF* operon was analyzed using a plasmid-borne *allF*′–′*lacZ* reporter and relevant regulator mutants. High expression of the *allF* operon under anaerobiosis with allantoin requires the local regulator AllS and the global regulator ArcA. EMSA confirmed the direct binding of AllS and ArcA to the promoter of *allF*. These findings indicate that the oxamate branch is directly regulated by the activator AllS, one of two local regulators (AllR and AllS) in the allantoin pathway. Furthermore, we identified ArcA as the activator of *allF* operon transcription under anaerobic conditions. Although allantoin degradation is known to occur only anaerobically, the regulator remains unidentified. Our findings demonstrate ArcA’s involvement. ArcA, the regulator of the *allF* operon, may also control other anaerobic genes in the allantoin cluster, directly or indirectly.

## Introduction

**Fig. 1:**
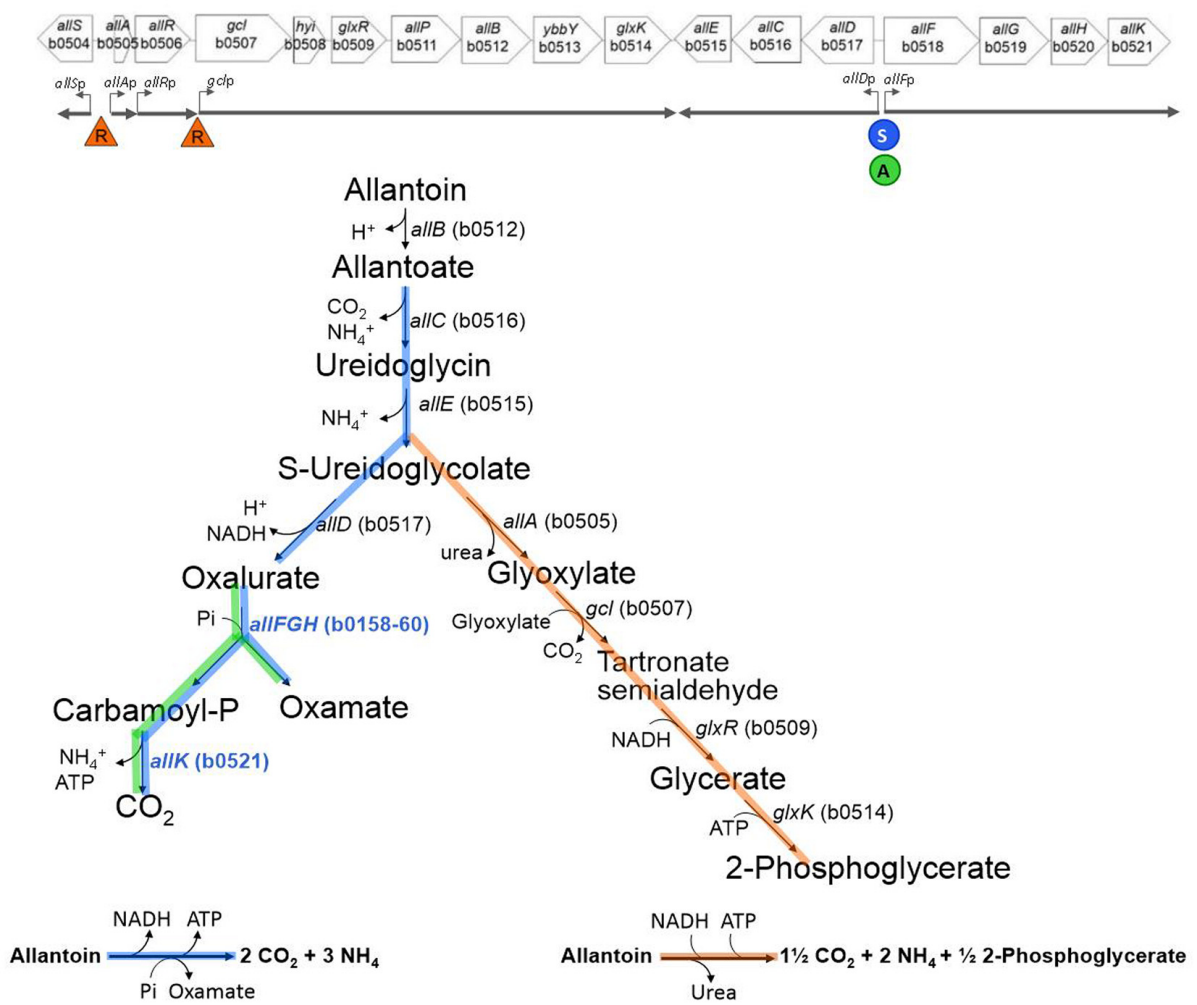
Allantoin degradation pathway and the associated gene cluster (ALL gene cluster) in *E. coli*. Allantoin is converted by allantonase (*allB*), allantoate amidohydrolase (*allC*), and ureidoglycine aminohydrolase (*allE*) into ureidoglycolate, which serves as a branch point. In the oxamate branch, ureidoglycolate dehydrogenase (*allD*) and oxamic transcarbamylase (allFGH) convert ureidoglycolate into oxamate and carbamoyl phosphate, the latter serving as a substrate for catabolic carbamate kinase (*allK*), which produces ATP and NH_3_. In the glycerate branch, ureidoglycolate hydrolase (*allA*) produces glyoxylate and urea; glyoxylate is further metabolized by glyoxylate carboligase (*gcl*), tartronate semialdehyde dehydrogase (glxR), and glycerate kinase (*glxK*) to 2-phosphoglycerate. NH_3_ yields per mole of allantoin are indicated below each branch of the pathway. Regulatory influences of AllR (R, orange), AllS (S, blue), and ArcA (A, green) are shown based on previously reported models and our working hypothesis. Detailed experimental evidence supporting these regulatory interactions is presented in the Results and discussed further in the Discussion.

*Escherichia coli* can utilize allantoin as its sole nitrogen source for anaerobic growth [1-3]. Allantoin (C_4_H_6_N_4_O_3_), a purine derivative containing four nitrogen atoms, is degraded to yield ammonia, which can then be assimilated as a nitrogen source. Initially, allantoin undergoes hydrolytic ring cleavage, hydrolysis, and oxidation catalyzed by three enzymes, AllB, AllC, and AllE. This releases two moles of NH_3_ and converts them into ureidoglycolate (C_3_H_5_N_2_O_4_^-^) [4-10] (Fig. 1). Ureidoglycolate is a metabolic branch point that can be converted into either oxalurate or glyoxylate [10]. The former is referred to as the oxamate branch, whereas the latter is the glycerate branch [3]. In the oxamate branch, ureidoglycolate is oxidized by AllD (ureidoglycolate dehydrogenase) to oxalurate (C_3_H_3_N_2_O_4_^-^) [3, 10]. Subsequently, oxalurate is converted into carbamoyl phosphate (CP) and the dead-end byproduct oxamate (C_2_H_2_NO_3_^-^) by oxamic transcarbamylase (OXTCase) [3, 11, 12], which has recently been found to be encoded by the genes *allFGH* [13]. The resulting CP is reutilized for the biosynthesis of nitrogen-containing compounds such as nucleotides and amino acids or is alternatively used to generate ATP by AllK (carbamate kinase), with an additional release of one NH_3_ [14].

In the glycerate branch, ureidoglycolate is converted by AllA (ureidoglycolate lyase) into glyoxylate and urea, but the urea ((NH_2_)_2_CO) is a waste product in urease-negative *E. coli* [8, 15]; see also Fig. 1. Glyoxylate is further metabolized by Gcl, GlxR, and GlxK into 2-phosphoglycerate, an intermediate of glycolysis, contributing to the carbon anaplerotic process [2, 16-19].

From a nitrogen perspective, degradation of one molecule of allantoin via the oxamate branch yields 3 NH_3_ and 1 oxamate, generating 1 ATP, whereas degradation via the glycerate branch yields 2 NH_3_, ½ 2-phosphoglycerate, and 1 urea, consuming 1 ATP.

The above allantoin degradation pathway is carried out by a set of 17 genes, termed the ALL gene cluster by Huynh and Stewart [3], arranged from *allS* to *allK* (b0504‒b0521) (Fig. 1, top). This cluster comprises three monocistronic genes (*allS*, *allA*, and *allR*) and three polycistronic operons: *gcl*‒*glxK* (*gcl* operon), *allD*‒*allE* (*allD* operon), and *allF*‒*allK* (*allF* operon). Among these six transcriptional units, the regulatory mechanisms of the first five have been studied, whereas less is known about the last unit, the *allF* operon (*allF*‒*allK*).

The repressor AllR and the activator AllS are two specific local regulators involved in controlling the expression of the ALL cluster genes. The repressor AllR binds to the promoter regions of *allS* and *allA* (*i.e.*, the control region of the divergent *allS*‒*allA* genes), as well as that of the *gcl* operon [20]. Glyoxylate acts as an inducer of AllR, whereas allantoin functions as a corepressor [3, 20]. As indicated in Fig. 1 (orange), AllR directly controls the genes involved in the glycerate branch.

AllS has been shown to activate the *allD* operon by binding to its promoter region [21]. Since *allD* and *allF* are divergently arranged and share a common control region. Moreover, the *allF* operon-encoded enzymes AllFGH (OXTCase) and AllK (carbamate kinase) function in the oxamate branch along with AllD, as illustrated in Fig. 1 (green). Therefore, the *allF* operon may also be under the control of AllS. However, the regulation of the *allF* operon has not been investigated, as the function of *allFGH* was only recently revealed to encode OXTCase [13]. In this study, we investigated the key regulatory factors that control the expression of the *allF* operon. Reporter gene fusion analysis using an *allF*'-'*lacZ* construct revealed that the two regulators, AllS and ArcA, activate the transcription of the *allF* operon. Subsequently, electrophoretic mobility shift assays (EMSAs) demonstrated that cell lysate overexpressing AllS or ArcA specifically bind to the upstream region of the *allF* operon. These findings indicate that allantoin degradation via the oxamate branch is regulated by both the local regulator AllS and the global regulator ArcA. Notably, although allantoin degradation exclusively under anaerobic conditions has been recognized for decades (since the 1960s), the underlying regulatory mechanisms have remained unclear. Here, we identify the global regulator ArcA as a key regulator acting at the final steps of this anaerobic pathway.

## Materials and Methods

### Bacterial Strains and Cultivation

Bacterial strains used in the fermentation experiments of this study were derivatives of *E. coli* MC4100 (Table 1). The relevant genotypes and sources of these strains are listed in Table 1. Cultivation was performed in nitrogen-deficient M9 minimal medium (_ND_M9) supplemented with 50 mM glycerol, 50 mM dimethyl sulfoxide (DMSO), and 30 mM allantoin [22]. Cultures were incubated at 37°C under anaerobic conditions in a gas mixture of 95% N_2_ and 5% H_2_. For the functional complementation of plasmids carrying target genes, isopropyl β-D-1-thiogalactopyranoside (IPTG; LPS Solution, Republic of Korea) was added at a final concentration of 0.2–1.0 mM.

### Construction of Gene-Deleted Strains and Plasmids

Genes were deleted by kanamycin resistance (kan^R^) gene replacement and transferred into the *E. coli* MC4100 genome using the P1 transduction method [23, 24]. P1 phage lysates were prepared from donor strains (Keio Collection) obtained from NBRP-*E. coli*, NIG, Japan (Table 1). Each lysate was transduced into MC4100 recipient strains and resulted in strains with *allS*, *arcA*, *allR*, *oxyR*, and *soxS* replaced by kan^R^, designated LMB162, LMB149, LMB161, LMB152, and LMB146, respectively (Table 1). In addition, kan^R^ markers were excised from LMB162 and LMB149 using the FLP recombinase-expressing plasmid pCP20, resulting in the marker-free strains MC4100Δ*allS* (LMB173) and MC4100Δ*arcA* (LMB174) (Table 1).

For the construction of pKNT25::*allS* and pKNT25::*arcA*, the genes were amplified from *E. coli* MG1655 genomic DNA using gene-specific primers (Table S1). The PCR products were cloned into the corresponding restriction sites of the pKNT25 backbone. These plasmids were transformed into *E. coli* DH5α cells and sequence-verified (Cosmogenetech Co., Ltd., Republic of Korea). The resulting constructs, pKNT25::*allS* and pKNT25::*arcA*, were designated pMB207 and pMB208, respectively (Table 1). Plasmid pNTR-SD::*allS* (p156#3) was obtained from NBRP-*E. coli*, NIG, Japan (Table 1).

### Analysis of Fermentation Products by HPLC

Fermentation products in culture supernatants were analyzed using a LaChrom Elite HPLC system (Hitachi High Technologies, Japan) equipped with an L-2130 pump, L-2350 column oven, L-2200 autosampler, and an Aminex HPX-87H ion-exclusion column (300 × 7.8 mm; Bio-Rad, USA). The mobile phase consisted of 2.2 mM H_2_SO_4_, delivered at a flow rate of 0.55 ml/min. Quantitative analysis was carried out using both a refractive index detector (L-2490) and a UV detector set at 210 nm (L-2400).

### Electrophoretic Mobility Shift Assay

The DNA fragments for EMSA were designed to be 60 base pairs in length. The DNA fragments used in EMSA were prepared by hybridizing the corresponding oligonucleotide sets listed in Table S3. Each oligonucleotide (100 μM) was hybridized in annealing buffer (100 mM NaCl, 50 mM HEPES, pH 7.4), with the temperature gradually lowered from 90°C to 10°C over 45 min.

The expression constructs pCA24N::*arcA* (JW4364-AM) and pCA24N::*allS* (JW0492-AM) (sourced from NBRP-*E. coli*, NIG, Japan) were used for ArcA and AllS expression, respectively (Table 1). Overexpression of ArcA and AllS was induced in *E. coli* strain AG1 harboring the corresponding plasmids by the addition of 0.2 mM IPTG at an OD_600_ of 0.3, followed by incubation at 30°C for 4 h.

To extract proteins, cell pellets were resuspended in lysis buffer (20 mM Tris-HCl, pH 8.3, 0.5% Triton X-100, 1 mg/ml lysozyme) and subjected to vigorous vortexing with acid-washed glass beads (G1145, Sigma-Aldrich, USA) for 30 min at 4°C. Cell debris was removed by centrifugation at 13,000 rpm for 20 min at 4°C, and the supernatant was collected. For the preparation of phosphorylated ArcA (ArcA~P), lysates of ArcA were phosphorylated by incubation at 30°C for 1 h in phosphorylation buffer containing 100 mM Tris-HCl (pH 7.0), 10 mM MgCl_2_, 125 mM KCl, and 50 mM lithium potassium acetyl phosphate (Sigma-Aldrich) [25]. Lysates for AllS and ArcA were quantified using the Bradford assay (Protein Assay Dye Reagent, 5000006; Bio-Rad) with bovine serum albumin as the standard.

Binding reactions for EMSA (20 μl total volume) were performed at room temperature for 30 min in reaction buffer containing 50 ng/μl poly(dI·dC), 0.05% NP-40, 2.5% glycerol, and 5 mM MgCl_2_ (LightShift^®^ Chemiluminescent EMSA Kit; Pierce Biotechnology, USA). DNA-protein complexes were separated on a 6% native polyacrylamide gel in 0.5× TBE buffer. The gel was transferred to a Biodyne B nylon membrane (Pall, USA) using a semi-dry transfer system (Trans-Blot Semi-Dry Electrophoretic Transfer Cell; Bio-Rad), and biotin-labeled DNA was detected using the ImageQuant 800 imaging system (Amersham, USA).

### Statistical Analysis

Statistical analyses were conducted using PASW Statistics 18 software (SPSS Inc., USA). Data were evaluated using unpaired two-tailed Student’s *t*-tests or one-way analysis of variance (ANOVA), as appropriate. When significant differences were detected by ANOVA, we used Duncan’s multiple range test for post hoc comparisons. A *p*-value of <0.05 was considered statistically significant.

## Results

### Anaerobic Growth with Allantoin Activates Expression of the *allF* Operon

**Fig. 2:**
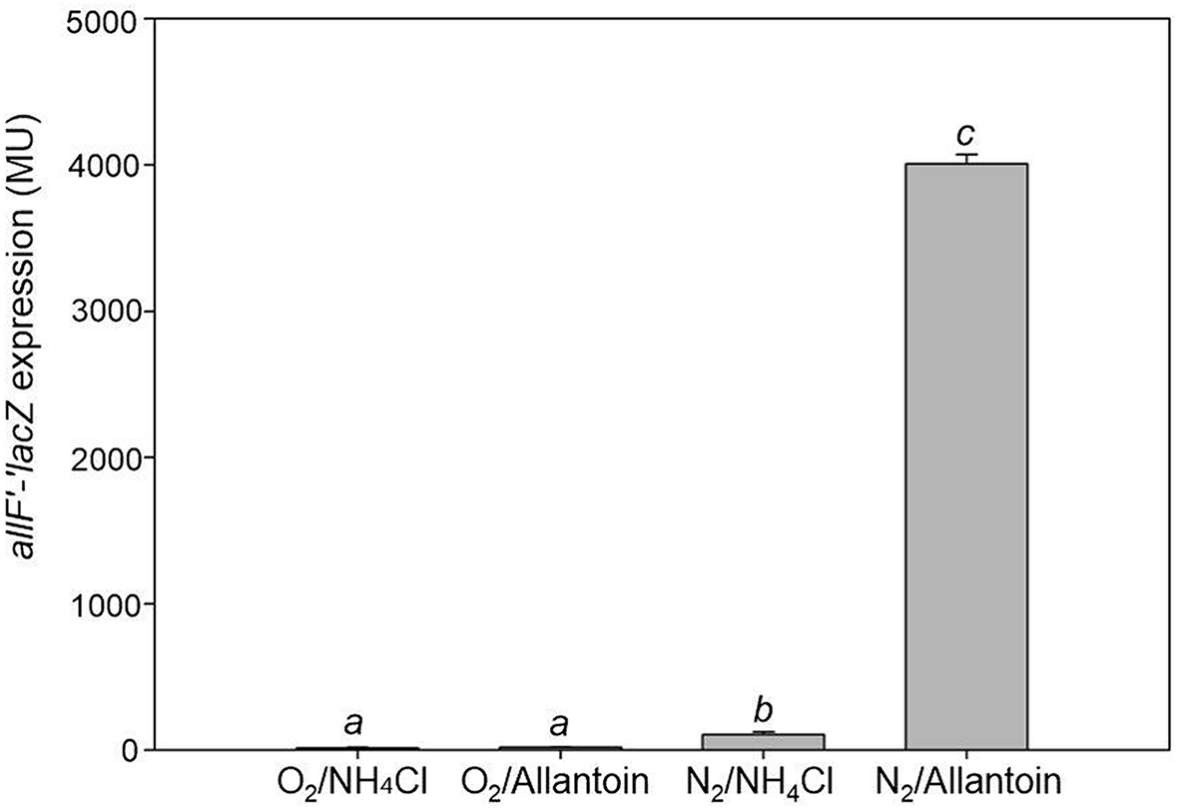
The expression of the plasmid-borne *allF*'-'*lacZ* reporter gene fusion in *E. coli* under different culture conditions. *E. coli* MC4100 harboring the plasmid pMB151 (*allF*'-'*lacZ*) were aerobically or anaerobically grown in nitrogendeficient M9 medium supplemented with glycerol (50 mM) and either allantoin (20 mM) or NH_4_Cl (20 mM) as the sole nitrogen source. For anaerobic culture, DMSO (50 mM) was provided as an electron acceptor. Different letters above the bars indicate statistically significant differences (*p* < 0.05). Values were determined from three replicates. Error bars indicate standard deviations.

Oxamic transcarbamylase (OXTCase) genes *allFGH* and the catabolic carbamate kinase (CK) gene *allK* form an operon *allFGHK* (*allF* operon) [14]. Their transcription was investigated by a β-galactosidase assay using *E. coli* MC4100 harboring the plasmid-borne *allF*'-'*lacZ* reporter fusion (pMB151) (Table 1). Cells were cultivated anaerobically or aerobically in _ND_M9 medium supplemented with glycerol (50 mM) as the carbon source and either ammonium chloride (NH_4_Cl, 20 mM) or allantoin (20 mM) as the sole nitrogen source. For anaerobic growth, DMSO (50 mM) was provided as an electron acceptor. Under aerobic conditions, *allF*'-'*lacZ* expression was not detected regardless of the nitrogen source, yielding 15 ± 3 Miller units (MU) with NH_4_Cl (O_2_/NH_4_Cl) and 19±2MU with allantoin (O_2_/allantoin) (Fig. 2). In contrast, under anaerobic conditions with allantoin as a nitrogen source, *allF*'-'*lacZ* expression was markedly elevated, reaching 4,007 ± 64 MU (N_2_/allantoin), which is more than 38-fold higher than with NH_4_Cl (105 ± 20 MU, N_2_/NH_4_Cl) (Fig. 2). These results demonstrate that the expression of genes *allFGHK*, which encode OXTCase and catabolic CK, enzymes of the oxamate branch that enable maximal ammonia release (Fig. 1), is dependent on both anaerobiosis and the presence of allantoin as sole nitrogen source, as previously reported [26].

### *In silico* Prediction of Transcriptional Regulators of the *allF* Operon

**Fig. 3:**
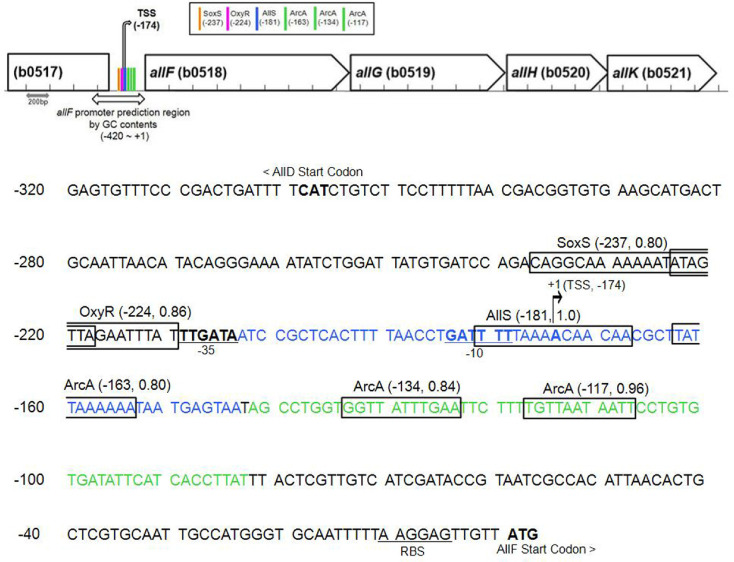
In silico prediction of transcriptional regulators for the *allF* operon. Prediction scores using VirtualFootprint (PRODORIC Virtual Footprint 4.0 server https://www.prodoric.de/vfp/). In the upper figure, binding positions of regulators and σ^70^ in intergenic regions are indicated: Numbers in parentheses indicate the central position of each predicted binding motif relative to the *allF* start codon. Prediction scores for regulators: ArcA, 0.96 (-117), 0.84 (-134), 0.80 (-163); AllS, 1.00 (-181); OxyR, 0.86 (-224); SoxS, 0.80 (-237). The prediction score for σ^70^ using iPro70-FMWin is 0.9978 and for σ^54^ using iPro54- PseKNC is 0.9121. The binding motifs predicted by the PRODORIC tool are indicated with boxes in the lower figure. Annotations for the underlined −10 and −35 promoter elements are based on information from the EcoCyc database [31]. Binding motifs on the nucleotide sequence of the *allF* promoter region in *E. coli* MG1655. The genomic sequence was obtained from NCBI (Accession No. NC_000913.3).

To predict transcriptional regulators that control the expression of the *allF* operon responding to allantoin and anaerobiosis, we employed the VirtualFootprint tool from the PRODORIC database. This tool enables *in silico* identification of potential transcription factor binding sites by scanning promoter regions using position weight matrices derived from experimentally validated binding motifs. When applying a high confidence score cutoff (≥0.8), four potential regulators, AllS, ArcA, OxyR, and SoxS, were predicted to bind upstream of the *allF* operon (*i.e.*, in the intergenic region between *allD* and *allF*) (Fig. 3, Table S2).

AllS was predicted with the highest confidence (score = 1.0) and could be the primary activator of *allF* under conditions where allantoin serves as the sole nitrogen source. In contrast, no binding site was identified for AllR, another regulator of the ALL cluster (Fig. 3, Table S2).

Regarding anaerobic regulation, ArcA was predicted to be involved, supported by three high-confidence binding sites (scores: 0.96, 0.84, and 0.80) and three additional lower-scoring sites (≥ 0.7) clustered nearby (Fig. 3, Table S2). Although Fnr is a major regulator of anaerobiosis, no binding site was detected. In addition, two potential binding sites for the oxidative stress regulators OxyR and SoxS were identified. Based on these predictions, we experimentally investigated the roles of AllS, ArcA, OxyR, and SoxS in regulating *allF* expression involved in the allantoin degradation pathway.

### Transcription of *allF* Operon Activated by AllS and ArcA

**Fig. 4:**
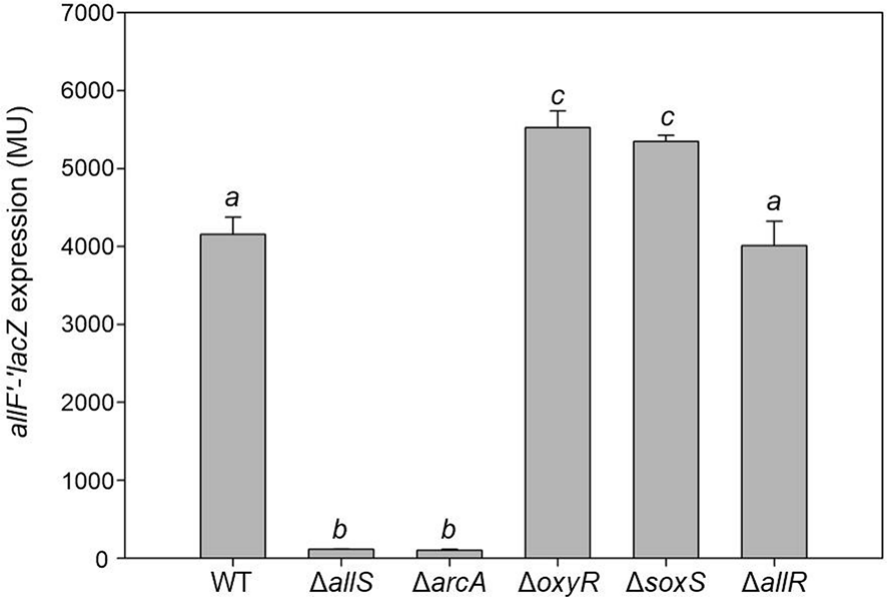
The expression of the plasmid-borne *allF*'-'*lacZ* reporter gene fusion in *E. coli* MC4100 wild-type and the regulator deletion mutants (Δ*allS*, Δ*arcA*, Δ*oxyR*, Δ*soxS*, and Δ*allR*). Cells were grown anaerobically for 48 h in nitrogen-deficient M9 minimal medium supplemented with glycerol (50 mM) and DMSO (50 mM), and allantoin (30 mM) as the sole nitrogen source. Different letters above the bars indicate statistically significant differences (*p* < 0.05), as determined by one-way ANOVA followed by Duncan’s multiple range test using SPSS PASW Statistics. Values were determined from three replicates. Error bars indicate standard deviations.

To examine whether the predicted regulators affect the expression of the *allF* operon, β-galactosidase activity was measured using the plasmid-borne *allF*'-'*lacZ* fusion (pMB151) in each regulator mutant of MC4100, Δ*allS*, Δ*arcA*, Δ*oxyR*, Δ*soxS*, and Δ*allR*. The cells were anaerobically grown in _ND_M9 medium supplemented with glycerol (50 mM), DMSO (50 mM), and with allantoin (30 mM) as the sole nitrogen source. Significant decreases in the β-galactosidase activity of *allF*'-'*lacZ* were observed in both Δ*allS* (115 ± 2 MU) and Δ*arcA* (102 ± 14 MU) mutants compared to the wild-type strain (4,155 ± 222 MU) (Fig. 4).

**Fig. 5:**
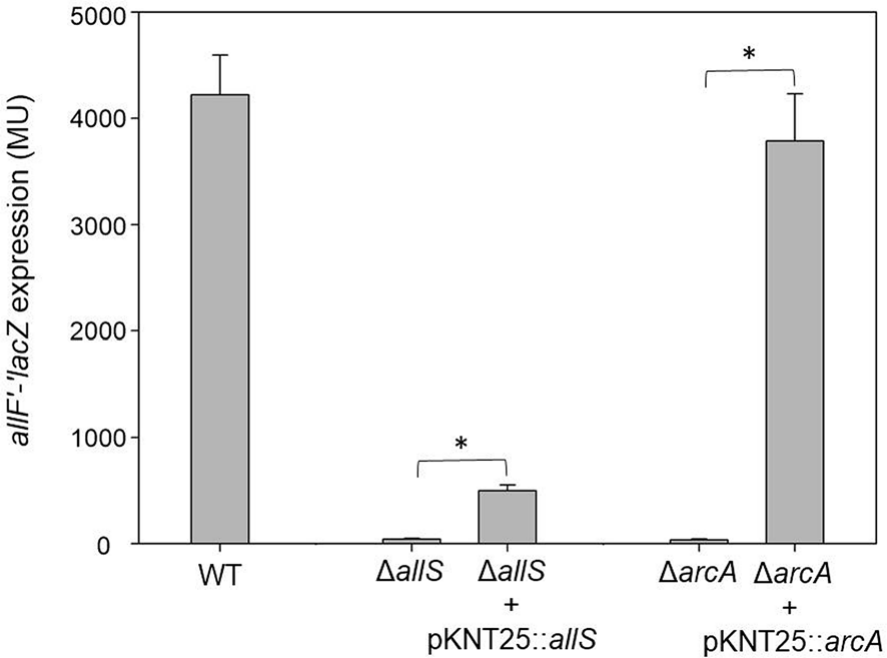
The β-galactosidase complementation of *allF*'-'*lacZ* in the Δ*allS* and Δ*arcA* mutant MC4100 strains using the plasmid-borne *allS* [pMB207(pKNT25::*allS*)] and *arcA* [pMB208 (pKNT25::*arcA*)]. Cells were anaerobically cultured for 72 h in nitrogen-deficient M9 minimal medium supplemented with glycerol (50 mM), DMSO (50 mM), and allantoin (30 mM). For induction, strains harboring pKNT25::*allS* or pKNT25::*arcA* were supplemented with 1.0 mM or 0.5 mM IPTG, respectively. Asterisks (*) indicate statistically significant differences (*p* < 0.05), as determined by unpaired two-tailed Student’s *t*-tests conducted using SPSS PASW Statistics. Values were determined from three replicates. Error bars indicate standard deviations.

To validate the regulatory roles of AllS and ArcA, β-galactosidase complementation assays were conducted by introducing pMB207 (pKNT25::*allS*) into the Δ*allS* mutant and pMB208 (pKNT25::*arcA*) into the Δ*arcA* mutant (Fig. 5). Complementation of the Δ*arcA* strain with pKNT25::*arcA* nearly restored the transcriptional activity (3,786 ± 446 MU) to wild-type levels (4,222 ± 373 MU). The complementation of *allS* with pKNT25::*allS* restored the expression in the Δ*allS* strain (from 42 ± 8 MU to 497 ± 55 MU), indicating partial but significant recovery. These results indicate that AllS and ArcA (possibly ArcA~P) function as key activators of *allF* transcription.

In contrast, deletion of *allR*, which was not predicted to bind to the *allF* promoter, had no significant effect (4,010 ± 311 MU), consistent with VirtualFootprint (Fig. 4). In the Δ*oxyR* (5,526 ± 214 MU) and Δ*soxS* (5,348 ± 79 MU) mutants, *allF* transcription was increased to some extent. The increased expression of *allF* is likely an indirect effect. Although OxyR and SoxS are deleted in the mutants, they are known activators involved in oxidative stress responses, which are typically inactive under anaerobic conditions. Therefore, it is unlikely that they directly regulate *allF*.

Taken together, the β-galactosidase assay results using each regulator mutant suggest that AllS and ArcA are key activators of *allF* transcription, whereas AllR, OxyR, and SoxS do not appear to play a direct regulatory role.

### The Oxamate Branch Depends on AllS and ArcA: Functional Validation

The *allF* operon encodes enzymes for the final steps of the oxamate branch (Fig. 1). We next investigated whether the candidate regulators affect allantoin degradation leading to oxamate formation. The mutant strains Δ*allS*, Δ*arcA*, Δ*oxyR*, Δ*soxS*, and Δ*allR* were anaerobically cultured for 48 h in _ND_M9 medium containing glycerol (50 mM), DMSO (50 mM), and allantoin (30 mM) as the sole nitrogen source. Allantoin degradation was analyzed using HPLC. In the wild-type strain, 11.1 mM allantoin was consumed and converted into 7.3 mM oxamate (Table 2). This indicates that allantoin was first converted to the branch point intermediate ureidoglycolate, of which 7.3 mM was further metabolized via the oxamate branch, while the remaining 3.8 mM was presumably directed to the glyoxylate branch. Conversely, in the Δ*allS* mutant, allantoin consumption was negligible (0.5 mM), and oxamate was not detected (Table 2). In the Δ*allS* mutant, genes *allFGHK* (*allF*'-'*lacZ* in Figs. 4 and 5) are almost not expressed. In addition, genes *allDEC* (*allD*'-'*lacZ* in Rintoul *et al*.) are also downregulated [21]. Consequently, allantoin degradation becomes impossible, resulting in poor growth of the Δ*allS* mutant when allantoin serves as the sole nitrogen source (Table S3).

In the Δ*arcA* mutant, allantoin consumption was also negligible (0.4 mM), and oxamate was not detected (Table 2). As shown by the *allF*'-'*lacZ* results in the Δ*arcA* background (Figs. 4 and 5), the expression of *allFGHK* genes was lost in the absence of ArcA, which explains the lack of oxamate production (Fig. 1). Here, the near-complete lack of allantoin consumption in the Δ*arcA* mutant suggests that ArcA is a key regulator the *allF* operon, and may also influence other genes wihin the allantoin cluster, either directly or indirectly. This is because allantoin can theoreticallybe metabolized via the glyoxylate branch which remains active even under aerobic conditions, without relying on the oxamate branch (Fig. 1).

Allantoin consumption in the Δ*oxyR* and Δ*soxS* mutants was comparable to that of wild-type (Table 2), consistent with the observation that the *allF*'-'*lacZ* expression remained largely unchanged (Fig. 4). Although the Δ*allR* mutant showed no change in *allF*'-'*lacZ* expression (Fig. 4), it consumed approximately twice as much allantoin (24.4 mM) as the wild-type and produced a large amount of oxamate (24.0 mM) (Table 2). This metabolic change is not due to direct regulation at the *allF* promoter, but rather could result from the loss of AllR, which represses the expression of the *allS*, *allA*, and *glc* operon (Fig. 1) [21]. Notably, the derepression of *allS*, a key activator of the *allF* operon, could be the major contributing factor to the increased oxamate branch, highlighting AllS and ArcA as key activators of this pathway.

To verify this, we conducted a complementation test to determine whether the blocked allantoin-to-oxamate conversion in Δ*allS* and Δ*arcA* mutants could be restored by introducing plasmids carrying either the *allS* or *arcA* gene. Introduction of p156#3 (pNTR-SD::*allS*) restored allantoin consumption in the Δ*allS* mutant from 0.4 mM to 24.2 mM, and oxamate production from 0 mM to 21.7 mM, both of which are comparable to wild-type levels (Table 3). Introduction of pMB208 (pKNT25::*arcA*) restored allantoin consumption in the Δ*arcA* mutant from 0.4 mM to 6.8 mM, and oxamate production from 0 mM to 2.4 mM. Although these values represent only partial restoration to wild-type levels, the recovery is nonetheless significant. Importantly, while this metabolic recovery was limited, the reduced *allF* transcription in the Δ*arcA* mutant was fully restored by pMB208 (pKNT25::*arcA*)(Fig. 5).

In summary, allantoin degradation and oxamate production via the oxamate branch were blocked in Δ*arcA* and Δ*allS* mutants, but restored upon complementation, confirming AllS and ArcA as key regulators.

### Binding of AllS and ArcA to the *allF* Promoter

**Fig. 6:**
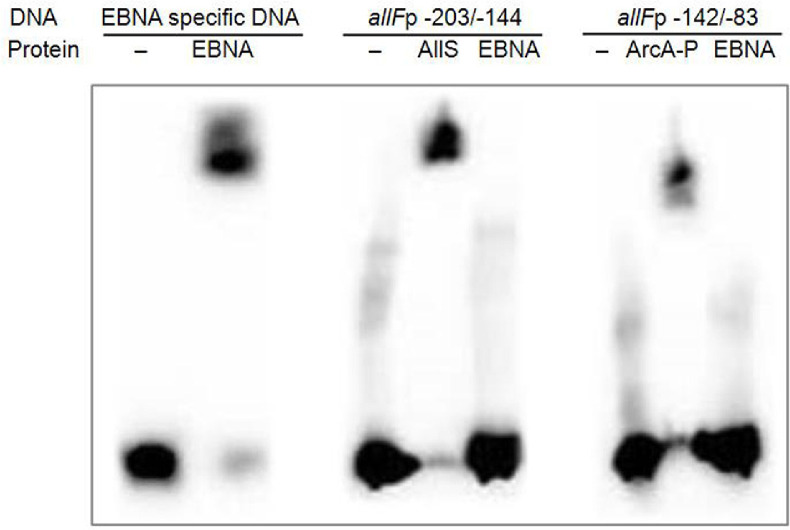
Electrophoretic mobility shift assay (EMSA) analysis of AllS and ArcA binding to the *allF* promoter region. Epstein-Barr Nuclear Antigen (EBNA) protein (1 Unit) and biotinylated EBNA-specific DNA (20 fmol) were used to validate the assay (left panel). Biotinylated DNA fragments (60 bps) containing the predicted binding motifs for AllS [***allFp*-203/-144**] and ArcA [***allFp*-142/-83**] (highlighted in blue and green in Fig. 3, respectively) were added (80 fmol) to binding mixtures containing 24 μg of crude lysate from strains harboring pCA245N::*allS* or 40 μg of phosphorylated crude lysate from strains harboring pCA245N::*arcA*, respectively. The EBNA protein was used as a negative control for AllS- and ArcA-specific binding.

The direct interaction of the regulators AllS and ArcA with the promoter region upstream of the *allF* operon was determined using EMSA. The two 60-bp DNA fragments were designated as fragment *allF*p-203/-144, which contains the predicted AllS-binding motif identified by Prodoric (located from -203 to -144 relative to the *allF* start codon [ATG]), and fragment *allF*p-142/-83, which includes the two highest-scoring ArcA-binding motifs (spanning positions -142 to -83 relative to the ATG) (Fig. 3). The DNA fragments were biotinylated at the 3' end using forward primers (Table S1). To prepare the protein, overexpression of AllS and ArcA was attempted using the vectors pCold, pET30b, and pCA24N. Overexpression was largely unsuccessful, though pCA24N yielded modestly improved expression. Attempts to perform EMSA with the purified protein were unsuccessful because of the very low yield or reduced activity (data not shown). Therefore, crude lysates prepared from cells expressing AllS or ArcA using pCA24N were used as an alternative in EMSA. To serve as a positive control in the EMSA, the EBNA protein and its specific biotinylated DNA probe were tested in parallel (Fig. 6, left panel). Incubation of the DNA fragment *allF*p-203/-144 with the AllS-expressing lysate (AllS ~35 kDa) resulted in markedly retarded mobility (Fig. 6, middle panel). In contrast, no retardation was observed with EBNA as a control, indicating specific binding of AllS to the *allF*p-203/-144 fragment.

For ArcA, EMSA was performed using ArcA-containing crude lysates (ArcA ~27 kDa), which were phosphorylated *in vitro* to generate ArcA-P, together with the DNA fragment *allF*p-142/-83. A clear mobility shift was also observed, though its intensity was slightly weaker than that observed with AllS (Fig. 6, right panel). This weaker shift may be due to incomplete *in vitro* phosphorylation of ArcA, which was necessary because all procedures for EMSA, from culture to assay, were conducted under aerobic conditions. Nonetheless, compared to the control, the mobility retardation indicates specific binding of ArcA-P to the *allF*p-142/-83 fragment.

**Fig. 7:**
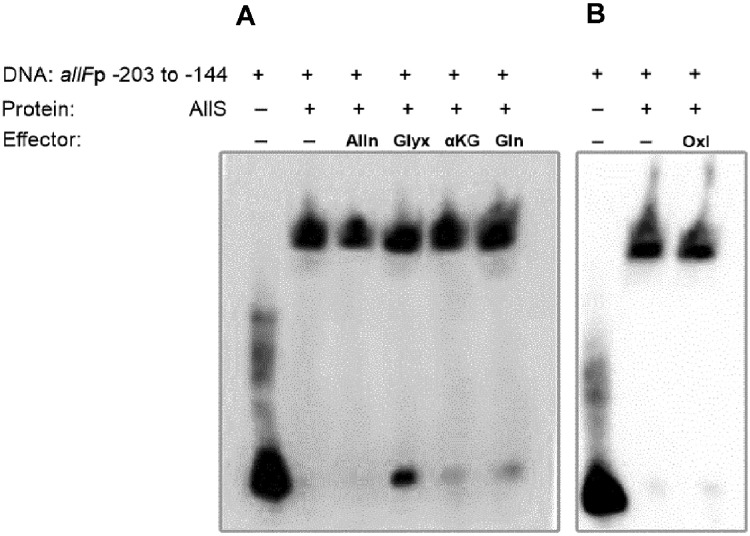
Electrophoretic mobility shift assay (EMSA) analysis of AllS with DNA fragment *allF*p-203/-144 in the presence of possible effector molecules. Biotinylated *allF*p-203/-144 DNA fragments (60 bps) containing the predicted AllS binding motif (highlighted in blue in Fig. 3) were added (80 fmol) to binding mixtures containing 24 μg of crude lysate from *E. coli* AG1 harboring pCA245N::*allS*. Alln (allantoin), Glyx (glyoxylate), αKG (α-ketoglutarate), and Gln (Lglutamine) (**A**), and Oxl (oxalurate) (**B**) were each added at a final concentration of 2 mM.

The influence of possible effector molecules on AllS-DNA binding was further examined in the presence of allantoin, glyoxylate, α-ketoglutarate, L-glutamine, and oxalurate, given that AllS belongs to the LysR-type transcriptional regulator (LTTR) family. Members of the LTTR family typically undergo conformational changes upon binding small-molecule effectors [27]. In the absence of these metabolites, AllS caused a clear mobility shift of the DNA fragment *allF*p-203/-144, and the extent of the shift remained unchanged by their presence (Fig. 7).

Altogether, EMSA results demonstrate that AllS specifically binds to the predicted motif located at −203 to −144, while ArcA-P binds to the region containing the top two predicted ArcA-binding motifs located at −142 to −83, both relative to the *allF* start codon. Furthermore, allantoin, glyoxylate, α-ketoglutarate, L-glutamine, and oxalurate did not affect AllS binding, indicating that they do not act as its effectors.

## Discussion

### AllS-Dependent Activation of the Oxamate Branch

Transcription of the ALL cluster genes is regulated by one of two specific local regulators, AllR or AllS, with only one acting directly at each promoter (Fig. 1, top). Our results using the *allF*'-'*lacZ* fusion show that *allFGHK* genes, which encodes oxamic transcarbamylase (OXTCase) and carbamate kinase (CK), is transcriptionally activated by AllS (Figs. 4 and 5). Together with previous findings by Rintoul *et al*. [21], which demonstrated AllS-dependent expression of *allDCE* using *allD*'-'*lacZ* fusion, this supports a broader role of AllS in regulating the entire oxamate branch, as illustrated by the blue-highlighted reactions in Fig. 1. However, allantoin does not act as an effector for AllS. Since AllS was shown to bind the promoter region regardless of the presence of allantoin, glyoxylate, α-ketoglutarate, L-glutamine, or oxalurate, its activation is unlikely to rely on these metabolites as direct effectors (Fig. 7). Given that allantoin and glyoxylate are typical ALL cluster effectors, α-ketoglutarate and L-glutamine reflect nitrogen status, and oxalurate is a direct substrate of the *allF*-encoded enzyme, these findings suggest that AllS-dependent activation may be achieved at the level of *allS* gene expression, as previously proposed [3, 21].

Rintoul *et al*. reported that *allS* expression is repressed by AllR [21]. Under nitrogen-replete conditions (20 mM NH_4_Cl), the addition of glyoxylate induces *allS* expression to levels comparable to those observed in a Δ*allR* mutant, consistent with the derepression of AllR by glyoxylate. Under nitrogen-limited conditions (0.5 mM NH_4_Cl), *allS* expression is further induced by approximately three-fold. These findings suggest that *allS* is regulated not only by AllR but also in response to nitrogen availability, which appears to be independent of NtrC and Nac [3, 21]. Switzer *et al*. discovered that allB plays a critical role in adaptation to long-term (24-h) nitrogen starvation, with allC and *allE* contributing to this process [28]. Although the initial starvation response was NtrC-dependent, the long-term response was independent of NtrC and involved the degradation of allantoin. There appears to be a regulatory mechanism responsive to nitrogen starvation, when allantoin is the sole available nitrogen source, that facilitates its utilization, possibly through increasing *allS* transcription. The mechanism underlying this nitrogen-responsive regulation warrants further investigation.

### Novel ArcA-Dependent Regulation of *allF* operon

Although allantoin degradation in *E. coli*, as in the originally studied *Streptococcus allantoicus* (*Carnococcus allantoicus*) [11], has long been known to occur exclusively under anaerobic conditions, the transcriptional regulators responsible for this anaerobic control have remained unidentified. In this study, we identify ArcA as a global regulator of the *allF* operon, revealing a previously unrecognized link between anaerobiosis and allantoin catabolism. Reduced *allF*'-'*lacZ* expression in the Δ*arcA* mutant and its full restoration by plasmid-borne *arcA* indicates that ArcA acts as an activator of the *allF* operon under anaerobic conditions (Fig. 5).

The ALL gene cluster consists of six distinct transcription units: *allS*p, *allA*p, *allR*p, *gcl*p, *allD*p, and *allF*p (Fig. 1). Among these, *allA*p, *allR*p, and *gcl*p are expressed under both aerobic and anaerobic conditions [21, 29], whereas *allD*p is expressed only under anaerobic conditions [21], and *allF*p is only anaerobically expressed as demonstrated in this study. Both *allD*p and *allF*p are transcriptionally inactive under aerobic conditions. In contrast, *allS*p is known to be expressed anaerobically [21], but its expression under aerobic conditions remains to be determined. The exclusive anaerobic expression of *allD*p and *allF*p suggests that these promoters are subject to transcriptional regulation, either through activation under anaerobic conditions or repression under aerobic conditions. In this study, we demonstrated that *allF*p is activated by ArcA under anaerobic conditions. Moreover, the *allF* and *allD* operons share a common intergenic promoter region (Fig. 1, top), within which multiple ArcA-binding motifs are predicted (Fig. 3, Table S2). The products of *allD* (ureidoglycolate dehydrogenase), *allF*-*allG*-*allH* (OXTCase), and *allK* (CK) act together in the oxamate branch. Therefore, it is possible that they are coordinately regulated by ArcA. Future studies will be needed to determine whether ArcA contributes to the anaerobic regulation of *allDp* expression.

Moreover, the Δ*arcA* strain is unable to utilize allantoin at all (Tables 2 and 3). This may be due to the absence of upstream enzymes such as AllE and AllC, which are encoded in the *allD* operon (Fig. 1). This suggests that ArcA not only regulates the oxamate branch but also plays a decisive role in determining whether anaerobic allantoin degradation proceeds. Under anaerobic conditions, the oxamate branch serves as the principal route for allantoin degradation. In contrast, the glycerate branch does not require anaerobic conditions [30]. Thus, the glycerate branch, unlike the oxamate branch, may serve as an alternative bypass route linking anaerobic allantoin degradation to central carbon metabolism via glyoxylate.

### Why Is Allantoin Degradation Restricted to Anaerobic Conditions?

The most immediate explanation, as discussed in the previous section, is that the expression of key enzymes required for allantoin degradation is strictly dependent on ArcA activation under anaerobic conditions. These enzymes include early catabolic enzymes AllE and AllC, as well as the oxamate branch enzymes AllD, AllFGH and AllK.

A second explanation may lie in the evolutionary adaptation of *E. coli* to the intestinal environment of its natural hosts, mammals. The mammalian gut is an anaerobic environment, and in primates, including humans, purine metabolites, such as urate and its breakdown product allantoin, can accumulate. This relates to a physiological trait; primates consume purine-rich foods but lack the enzymes required to further degrade uric acid. In such an environment, the ability to catabolize allantoin and extract nitrogen may confer a selective advantage for enterobacteria, particularly under anaerobic and nitrogen-limited conditions. Therefore, its restriction to anaerobic conditions likely reflects adaptation to environments where this pathway offers the greatest advantage.

## Figures and Tables

**Table 1:** Strains and plasmids used in this study.

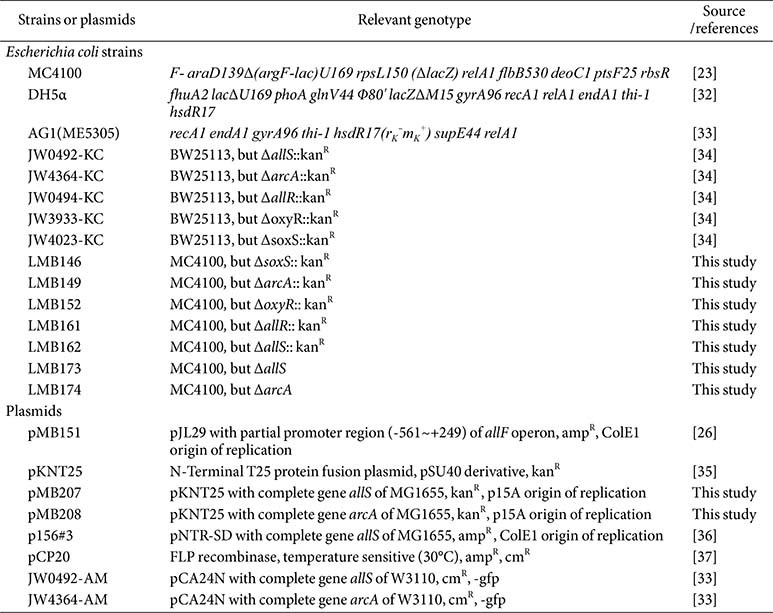

**Table 2:** HPLC analysis of anaerobic allantoin degradation in strains with deletion of specific regulatory genes.

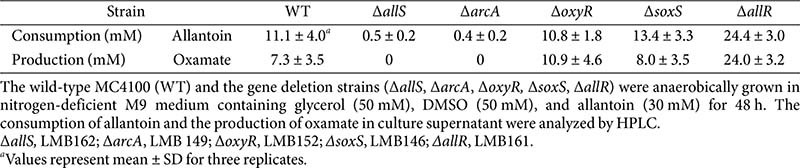

**Table 3:** HPLC analysis of anaerobic allantoin degradation in gene deletion strains Δ*allS* (LMB 173) and Δ*arcA* (LMB174) and their complementation with plasmid-borne *allS* (pNTR-SD::*allS*, p156#3) or *arcA* (pKNT25::*arcA*, pMB208).